# *Snrnp25* is a candidate for the peri-implantation lethal phenotype of the *Hba* deletions

**DOI:** 10.1007/s00335-025-10133-z

**Published:** 2025-05-21

**Authors:** Ana María Velásquez-Escobar, Andrew E. Hillhouse, Terry Magnuson, David W. Threadgill

**Affiliations:** 1 Department of Cell Biology and Genetics, Texas A&M University, College Station, TX 77843 USA; 2 Interdisciplinary Graduate Program in Genetics and Genomics, Texas A&M University, College Station, TX 77843 USA; 3 Department of Genetics, University of North Carolina, Chapel Hill, NC 27599 USA; 4 Department of Nutrition, Texas A&M University, College Station, TX 77843 USA

**Keywords:** Embryonic lethal, Deletion complex, Mouse model, Thalassemia

## Abstract

Mutations in adult hemoglobin alpha genes in humans lead to blood disorders commonly known as α-thalassemia. In search of a mouse model for this disease, mutagenesis screens have identified several deletions that resemble these phenotypes. The *Hba*^*b2(th)*^ deletion, in particular, replicates the characteristics of alpha-thalassemia minor in heterozygous mice but presents a homozygous embryonic lethal phenotype. Previous analyses of *Hba*^*b2(th)*^ mice suggested that the deletion affects both *Hba* genes (*Hba-a1* and *Hba-a2*) and considered epidermal growth factor receptor (*Egfr*) or rhomboid 5 homolog 1 (*Rhbdf1*) to be responsible for the embryonic lethality. Molecular analysis of *Hba*^*b2(th)*^ revealed a deletion spanning a 1 cM region of mouse chromosome 11. Importantly, the *Hba*^*b2(th)*^ deletion does not extend to *Egfr*, indicating that the observed lethality of homozygous embryos is not due to the loss of *Egfr*. Sequence analysis of the *Hba*^*b2(th)*^ deletion showed that the *Hba-a2* gene is not deleted, but the lack of expression is likely due to the disruption of upstream regulatory regions. Furthermore, we identify *Snrnp2*5, which codes for the small nuclear ribonucleoprotein 25 (U11/U12), as the candidate gene most likely responsible for the peri-implantation lethality of *Hba*^*b2(th)*^ homozygous mice. These findings enhance the understanding of the genetic mechanisms underlying α-thalassemia and provide insights into novel genes essential for early mammalian development.

**Supplementary Information:**

The online version contains supplementary material available at 10.1007/s00335-025-10133-z.

## Introduction

Alpha-thalassemia is a genetic disorder produced by low or absent expression of the α-globin chain subunit of hemoglobin (Weatherall and Clegg 1972). In both humans and mice, there are two adjacent adult hemoglobin alpha genes (*HBA1* and *HBA2* in humans; *Hba-a1* and *Hba-a2* in mice), along with an embryonic version (Whitney and Russell 1980), all tightly linked on human chromosome 16 (HAS16) and mouse chromosome 11 (MMU11), respectively. Humans who are heterozygous for α-globin mutations have α-thalassemia, presenting with mild histological defects of the erythrocytes (Nathan and Gunn 1966). Homozygosity for mutations in *HBA1* and *HBA2* can result in α-thalassemia major, which leads to a complete loss of α-globins and is lethal between 30 and 40 weeks of gestation (Taylor et al. 1974). Affected individuals exhibit the fatal congenital disorder known as hydrops fetalis. Hematologic defects associated with alpha-thalassemia minor arise from a reduction in the normal 1:1 subunit ratio of α-globin to β-globin, the latter being encoded by a separate unlinked gene cluster, due to mutations in two of the four alleles of the *HBA* genes.

With the goal of identifying a mouse model for α-thalassemia, radiation and chemical mutagenesis screens were undertaken. Two x-ray-induced, *Hba*^*b2(th)*^ and *Hba*^*b3(th)*^ (Russell et al. 1976a), and one triethylenemelamine-induced, *Hba*^*th−J*^(Hendrey et al. 1995; Whitney and Russell 1978), mutations were recovered. Molecular characterization of these animals revealed that they were heterozygous for deletions of the *Hba* gene cluster (Whitney et al. 1981). Matings between heterozygous animals produced no homozygous pups, indicating that homozygosity for any of the three deletions is embryonic lethal. However, closer examination of earlier embryonic stages revealed some unexpected results. A retrospective analysis of embryos from *Hba*^*b 2 (th)*^/+ crosses showed that the putative homozygous embryos were dying shortly after initiating implantation at embryonic day (E) 5.5 to 6.5(Popp et al. 1980). Since hemoglobins are not necessary until E8.0 when the blood islands of the yolk sac begin producing red cells (de Aberle 1927), this observation suggested that the deletions around the *Hba* complex not only removed the *Hba* genes but also deleted or impacted other genes involved in peri-implantation development. Attempts to create homozygous mutant ES cell lines were unsuccessful, further supporting the notion that the deletions have removed or affected other loci (Behzadian et al. 1993). A subsequent study implicated N-methylpurine-DNA glycosylase (*Mpg*) and rhomboid 5 homolog 1 (*Rhbdf1*, previously called *Dist 1*) as candidate genes for the peri-implantation lethality of the *Hba* deletion homozygotes, based on their expression during peri-implantation and their loss in the *Hba*^*th− J*^ deletion (Hendrey et al. 1995). In another study, liver preparations from *Hba*^*b 3 (th)*^ deletion heterozygous mice were found to bind half the epidermal growth factor (EGF) compared to preparations from their normal littermates, partly due to lower levels of epidermal growth factor receptor (*Egfr)* mRNA (Behzadian et al. 1990).

Based on these data, it was hypothesized that the deletions also affected *Egfr*, which maps only six centimorgans (cM) proximal to *Hba (*Silver et al. 1985). Furthermore, similar to *Hba* deletion homozygotes, embryos homozygous for *Egfr*^*tm1Mag*^, a targeted null allele for *Egfr*, can die during peri-implantation, depending on genetic background (Threadgill et al. 1994). Since x-rays can produce deletions covering several centimorgans, the phenotype originally reported for the *Hba* deletions could be attributed to a loss of *Mpg*, *Rhbdf 1*, or *Egfr*. Our study performed a molecular analysis of the deletions and identified the gene coding for small nuclear ribonucleoprotein 25 (U11/U12) (*Snrnp25*) as most likely responsible for the peri-implantation lethality of *Hba* deletion homozygotes.

## Materials and methods

### Animals

DNA samples from mice carrying *Hba*^*b3(th)*^ and mice carrying *Hba*^*b2(th)*^ were obtained from the Oak Ridge National Laboratory and DNA samples from mice carrying *Hba*^*th−J*^ from Medical College of Georgia. The original *Hba*^*b2(th)*^ deletion was generated on SEC/R1 mice (Russell et al. 1976a). B6.SEC-*Hba*^*b2(th)*^ mice were crossed to CAST/EiJ (CAST) to generate an F1 hybrid for increased polymorphisms between the wildtype and *Hba*^*b2(th)*^ carrying MMU11.

Animals were maintained in accordance with the Institution Animal Care and Use Committee (IACUC). They were housed at 22 °C under a 12-h light cycle. α-thalassemia phenotypic typing using blood from *Hba*^*b2(th)*^ mice was performed as described in (Russell et al. 1976b).

### PCR assays and amplicon sequencing

PCR-based SSLP assays were carried out using primers publicly available in The Jackson Laboratory’s Mouse Genome Informatics database (MGI, https://www.informatics.jax.org/) (Baldarelli et al. 2024). PCR assays aimed at narrowing deletion edges were designed using the NCBI Primer Blast tool (https://www.ncbi.nlm.nih.gov/tools/primer-blast/index.cgi), and the primers were synthesized by Integrated DNA Technologies (Table 1). Premium PCR sequencing of amplicons from potentially deleted genes was conducted by Plasmidsaurus using Oxford Nanopore Technology, accompanied by custom analysis and annotation. To establish whether a gene was deleted, a FASTA file for each amplicon was generated based on data from the per-base file provided by Plasmidsaurus. If a single base had approximately 50% of reads indicating two different nucleotides, the FASTA file would contain the IUPAC Ambiguity letter corresponding to that combination. Amplicon sequences from C57BL6/J (B6) (GRCm39) and CAST (from the MGI Multi-Genome Viewer) reference sequences, as well as from (B6.SEC-*Hba*^*b2(th)*^ x CAST)F1 mice, were aligned on Benchling (https://www.benchling.com/) using MAFFT (Katoh and Standley 2013). The region was classified as deleted if the (B6.SEC-*Hba*^*b2(th)*^ x CAST)F1 was found to be hemizygous in loci where the (B6.SEC-*Hba*^*wt*^ x CAST)F1 amplicon was heterozygous (Supplementary Data).

**Table 1:** PCR primers used for analysis of *Hba* deletions

Marker/Gene	F Primer	*R* Primer	MGI ID
D11Mit53	GTGGATACAGAGTGGATACAGGG	GTAACCAAGATGCAAGGGGA	89,133
D11Mit172	TGCCTAATTTAATTTGGATGGG	TGAGGTGTGTGTCTTGCCTC	89,058
D11Mit173	AAGTGACATATGGATTCCTGGG	TCAAAGTGGGTATGTGTCATCC	89,059
D11Mit186	AAAACACATTTACATGCATGGTG	TGTGTGCACTTAAGCCCTGA	89,072
D11Mit171	AAAGCATATGTCAAAAAACAAAACA	CACAGTCCTACCAGTCCATAAGC	89,057
D11Mit77	GTATTCAAATGACTTCTGCCTGG	TTGAAATGGTCTTCAAGTGGC	89,156
D11Mit215	CATTGGGGGATATGAAGTGG	CTTGTAAGCAGGACAAAATTTGG	101,276
D11Mit268	CCCCAGAACTCACATCAGGT	ATTCATTGTTGCCAGCAGG	101,223
D11Mit135	GCATCTGCAGAGGAGGTTTC	AAATGGAATTTAGTAAATGGGAAGG	89,018
D11Mit205	GGCAGAGTCTAGTCTGATATCTTGG	CAGTGCACAGCCAGGTTG	89,094
*Egfr* ^*tm1Mag*^	CTCAGCCAGATGATGTTGAC	CCTCGTCTGTGGAAGAACTA	95,294
*Cpeb4*	CCAGCAAGTTGGCTCAGCAGGT	AGGACTGTCCAGGAGCCAGTGA	1,914,829
*Bod1*	TGGCTCATGGCCACTGAAAT	CTGTGGCCTGAAGATGTGGT	1,916,806
*Hba-a2*	CATGGTGCTCTCTGGGGAAG	AGAGGTACAGGTGCAAGGGA	96,016
*Hbq1b*	ATGTCGGAATCTACGCGACC	TACTGGACGCGGGGAGTAT	3,613,460

### Whole genome sequencing and bioinformatics analyses

Tail samples from (B6.SEC-*Hba*^*b2(th)*^ x CAST)F1 mice were collected and snap-frozen. DNA extraction, sequencing library preparation using an Illumina TruSeq DNA library prep kit, and whole genome sequencing on an Illumina HiSeq 2500 was performed by the Texas A&M AgriLife Research Genomics and Bioinformatics Service. All bioinformatic analyses were conducted using the computing resources provided by Texas A&M High-Performance Research Computing. Fastq files were trimmed using trimmomatic (Bolger et al. 2014), and read quality was evaluated with FastQC (Andrews 2010). Filtered reads were mapped to the soft-masked GRCm39 mouse reference genome using Burrows-Wheeler Alignment (Li 2013). Mapped reads were then pre-processed following the GATK Best Practices workflow (DePristo et al. 2011). Genetic variants between B6 and CAST mouse strains were accounted for during this process. Finally, LUMPY (Layer et al. 2014) was used to call structural variant differences between the samples.

## Results

### The *Hba*^*b2(th)*^ deletion does not include *Egfr*

**Fig. 1:**
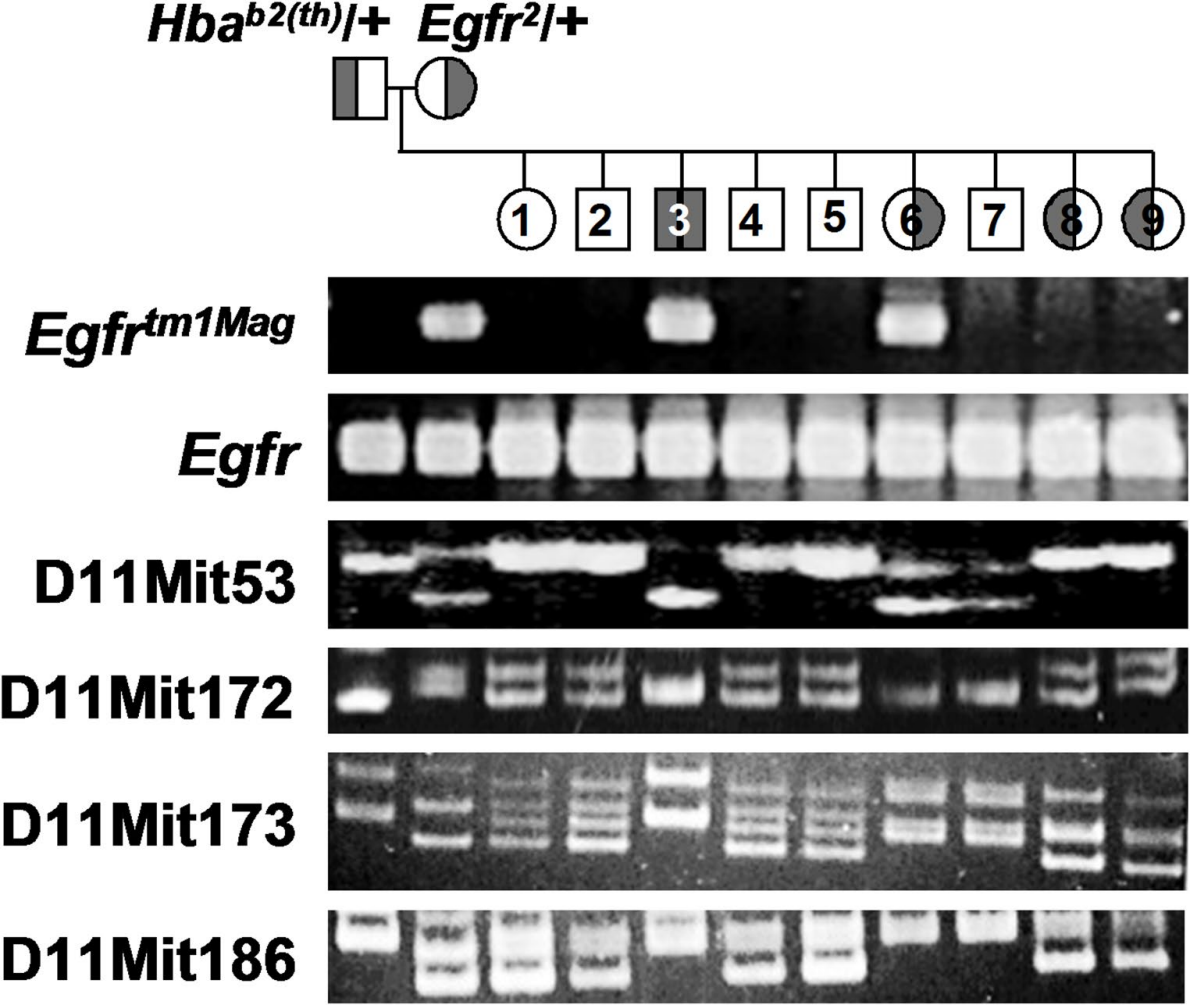
Genetic cross and ethidium bromide-stained gels used for complementation analysis. The individual relationships are shown at the top along with the genotypes of the parents. Individuals with their left half shaded carry the *Hba*^*b2(th)*^ deletion, while those with their right half shaded carry the *Egfr*^*tm1Mag*^ null-allele. Individual progeny are numbered. The locus names are on the left. Mice heterozygous at some SSLP loci, like D11Mit173 and D11Mit186, often show anomalous heteroduplex structures after PCR, as seen on progeny 1, 2, 4, 5, 8, and 9 (Routman and Cheverud 1994)

Since homozygosity for the *Hba*^*b2(th)*^ deletion and the genetic deletion of *Egfr*, which are syntenic on MMU11, can result in peri-implantation lethality depending on the genetic background, a genetic cross between *Hba*^*b2(th)*^/+ and *Egfr*^*tm1Mag*^/+ mice was performed to test for complementation. DNA from the resulting progeny was screened using PCR for *Egfr* alleles (Threadgill et al. 1994) and the simple sequence length polymorphism (SSLP) D11Mit53. Progeny number three inherited the maternally derived *Egfr*^*tm1Mag*^ null allele along with a paternally derived wild-type (wt) *Egfr* allele, in addition to the paternally derived *Hba*^*b2(th)*^ deletion chromosome (Fig. 1). This conclusion stems from the fact that progeny number three only possesses the lower band (maternal allele) and no higher band (paternal wild-type allele) for D11Mit53. Progeny number eight and nine also inherited the *Hba*^*b2(th)*^ deletion chromosome but possess the larger maternal allele and two wild-type *Egfr* alleles. Had *Hba*^*b2(th)*^ encompassed the EGFR locus, progeny number three would not have survived fetal development, as *Egfr* null animals are lethal. Therefore, *Egfr* is not part of the *Hba*^*b2(th)*^ deletion.

### Mapping *Hba* deletions on chromosome 11

**Fig. 2:**
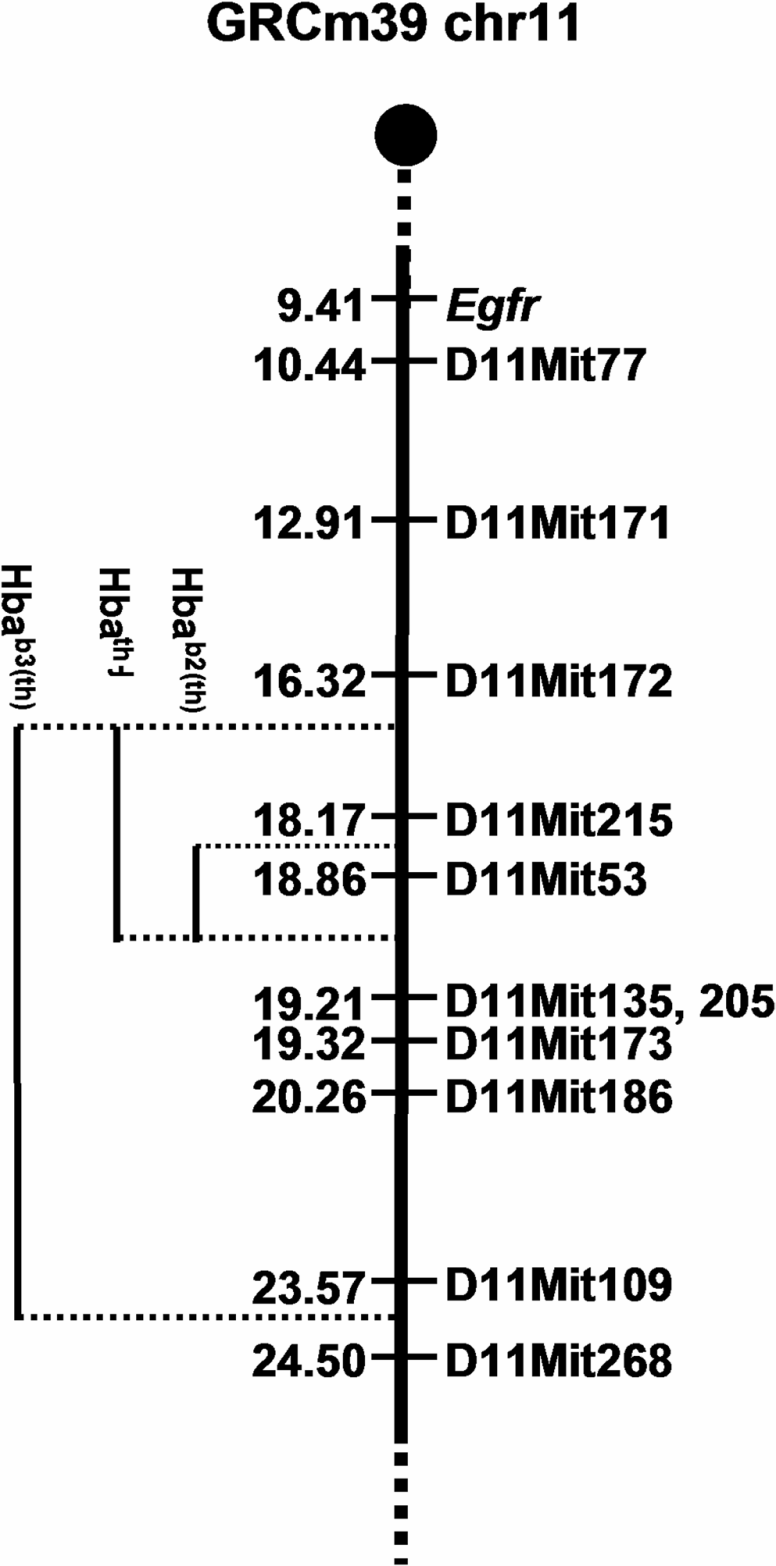
Genetic map of the *Hba* region of MMU 11. Genetic distances (in cM) from the centromere are on the left, and individual loci tested are on the right. The regions covered by each *Hba* deletion are shown on the left

To better estimate the genetic length of the *Hba* deletions, SSLP markers mapping to this region (Copeland et al. 1993; Dietrich et al. 1996) were screened by PCR. Markers spanning a 20 cM region around *Hba* indicated that the *Hba*^*b2(th)*^ deletion spans less than 1.04 cM on the genetic map, while the other two deletions (*Hba*^*b3(th)*^ and *Hba*^*th−J*^) are much larger (approximately 8.2 cM and 2.9 cM, respectively). For *Hba*^*b3(th)*^ and *Hba*^*th−J*^, all tested markers proximal to D11Mit172 are present (D11Mit172, D11Mit171, and D11Mit77), while the next distal marker, D11Mit215, is absent. This contrasts with *Hba*^*b2(th)*,^ in which D11Mit215 is not deleted (Fig. 2). Markers distal to D11Mit268 in *Hba*^*b3(th)*^, and D11Mit135 and D11Mit205 in *Hba*^*th−J*^ are present on the deletion chromosomes. Other markers at these two loci were uninformative due to a lack of polymorphism between the two parental strains.

To estimate the length of the *Hba*^*b2(th)*^ deletion, which is the shortest of the *Hba* deletions, and to confirm the previous estimate of the deletion size, offspring from interspecific crosses between the *M. m. musculus* (B6.SEC) deletion and *M. m. castaneous* (CAST) were generated to increase genetic differences. For the *Hba*^*b2(th)*^ deletion, marker D11Mit53 (mapping to 18.86 cM on GRCm39) is deleted, while D11Mit172 (16.32 cM), D11Mit173 (19.32 cM), and D11Mit186 (20.262 cM) are present on the deletion chromosome (Figs. 1 and 2).

### Physical length estimation of *Hba*^*b2(th)*^

**Fig. 3:**
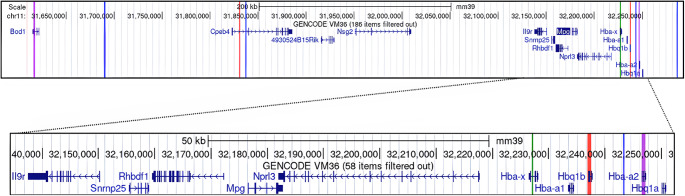
Chromosome visualization of *Hba*^*b2(th)*^ deletion. Screenshot from the UCSC Genome Browser mm39. Blue lines: Lumpy output candidate deletion edges at MMU11:31’690,316 or 31’836,857 (upstream), and chr11:32’243,280 or 32’286,351 (downstream). Red lines: regions confirmed deleted through amplicon sequencing, *Cpeb4* intron 1, and *Hbq1b*. Green line: regions confirmed deleted through SSLP analysis, D11Mit53, *Hba-x*. Purple lines: Regions confirmed not deleted through amplicon sequencing, *Bod1* intron2/exon3, and *Hba-a2*

Whole-genome sequencing of DNA from (B6.SEC-*Hba*^*b2(th)*^ x CAST)F1 mice allowed for further estimation of the *Hba*^*b2(th)*^ deletion boundaries. Haplotype analysis by Lumpy provided four candidate deletion edges at mm39-MMU11:31’690,316 or 31’836,857 (upstream), and mm39-MMU11:32’243,280 or 32’286,351 (Fig. 3, blue lines) surrounding the hemoglobin alpha complex. In-depth PCR-based analysis and sequencing of potentially deleted genes revealed that the upstream edge of the deletion lies between biorientation of chromosomes in cell division 1 (*Bod1*) and cytoplasmic polyadenylation element binding protein 4 (*Cpeb4*), while the downstream edge lies between hemoglobin, theta 1B (*Hbq1b*) and *Hba-a2* (Fig. 3, purple and red lines). This shows that the *Hba*^*b2(th)*^ deletion is between 31.7 Mb and 32.2 Mb, encompassing eleven known protein-coding genes.

## Discussion

### The alpha-thalassemia phenotype in *Hba*^*b2(th)*^ mice is not due to deletion of both *Hba* genes

Previous studies analyzing DNA using Southern blots and amino acid sequences of *Hba*^*b2(th)*^ heterozygous mice concluded that the deletion spans both the *Hba-a1* and *Hba-a2* genes (Popp et al. 1979; Whitney et al. 1981). The methods used in the earlier analysis have sensitivity limitations in amino acid detection and lack DNA loading controls. Phenotypic analysis of *Hba*^*b2(th)*^ heterozygous mice shows similar hematological alterations to those in humans with α-thalassemia minor (Popp and Enlow 1977; Whitney et al. 1981), which is a result of deletions or other mutations in two of the four alpha-globin alleles (either in *cis* or *trans*) (Tesio and Bauer 2023). We demonstrate here that the coding sequence of *Hba-a2* is not deleted. Therefore, the lack of expression of this gene is likely due to a disruption in its regulation. Considering the closest gene upstream of *Hba-a2*, *Hbq1b*, is deleted, it is reasonable to hypothesize that the deletion of the *Hba-a2* promoter region causes the lack of expression. The deletion edges indicated by Lumpy analysis suggest a boundary approximately 3 kilobases (kb) upstream of the first exon of *Hba-a2*. This region contains histone modifications and transcription factor binding sites that are likely important regulators of the expression of this gene (Oudelaar et al. 2021). Furthermore, the expression of alpha globin genes is highly regulated by a super-enhancer region within an intron of *Nprl3* (Hay et al. 2016), which lies within the deletion boundaries. Our results suggest that the phenotype observed in *Hba*^*b2(th)*^ mice resembles more closely that of human α-thalassemia cases involving mutations in the regulatory region rather than mutations in the alpha globin genes themselves (Hatton et al. 1990; Kalle Kwaifa et al. 2020; Sollaino et al. 2010).

### Gene(s) responsible for *Hba* deletion homozygous peri-implantation lethality

After confirming that *Egfr* is not deleted and is therefore not responsible for peri-implantation lethality, we created a list of all protein-coding genes within the *Hba*^*b2(th)*^ deletion using resources available from the MGI database (https://www.informatics.jax.org/). Gene-specific knockout phenotypes annotated by the International Mouse Phenotyping Consortium (IMPC, http://www.mousephenotype.org(Groza et al. 2022),, along with other phenotypes reported in the literature, indicated that *Snrnp25* is the most likely candidate gene for the peri-implantation lethal phenotype observed in *Hba*^*b2(th)*^ homozygous embryos (Table 2). Cells annotated as “Not significant” represent genes for which knockout alleles were generated and assessed by IMPC, and no mortality phenotype was observed.

**Table 2:** Protein-coding genes deleted in the *Hba*^*b2(th)*^ chromosome

Start	End	Symbol	Name	IMPC report	Other phenotypes
31,822,211	31,885,634	*Cpeb4*	Cytoplasmic polyadenylation element binding protein 4	Early adult lethality	
31,915,592	31,929,651	*4930524B15Rik*	RIKEN cdna 4930524B15 gene	Not significant	
31,950,463	32,009,202	*Nsg2*	Neuron specific gene family member 2	Not significant	
32,137,541	32,150,279	*Il9r*	Interleukin 9 receptor	Not significant	
32,155,415	32,158,984	*Snrnp25*	Small nuclear ribonucleoprotein 25 (U11/U12)	Not phenotyped	
32,159,585	32,172,300	*Rhbdf1*	Rhomboid 5 homolog 1	Early adult lethality	
32,176,505	32,182,700	*Mpg*	N-methylpurine-DNA glycosylase	Not significant	
32,181,963	32,217,707	*Nprl3*	Nitrogen permease regulator-like 3	Not phenotyped	E15.5-18.5 lethality (Kowalczyk et al. 2012)
32,226,600	32,228,116	*Hba-x*	Hemoglobin X, alpha-like embryonic chain in Hba complex	Not phenotyped	Embryonic lethal in some genetic backgrounds, viable in others (Leder et al. 1997)
32,233,672	32,234,486	*Hba-a1*	Hemoglobin alpha, adult chain 1	Not phenotyped	Homozygous knockout viable (Leder et al. 1997)
32,236,965	32,237,784	*Hbq1b*	Hemoglobin, theta 1B	Not phenotyped	

Of all the genes within the deleted region, only two lack previously reported knockout phenotypes: *Hbq1b* and *Snrnp25*. The role and expression pattern of *Hbq1b* are not well studied in mouse models. Expression analysis indicates this gene is first expressed during mouse gastrulation (Baldarelli et al. 2021). Similarly, human analyses show that this gene begins to be expressed during embryonic development after the expression of the fetal HBA subunit, HBZ (ortholog to mouse *Hba-x*), starts to decrease. The gradual switch from fetal to adult hemoglobin occurs during E10-14 of mouse development (Albitar et al. 1992; Fantoni et al. 1969), long after the lethality of *Hba*^*b2(th)*^ homozygous embryos.

*Snrnp25* codes for a core protein in the U12-dependent (minor) spliceosomal complex (Will et al. 2004). While only found in a very small proportion of genes in the mouse genome, U11/12-type introns are enriched in genes involved in genetic information processing (Turunen et al. 2013). Additionally, mutations in components of the minor spliceosome have been shown to be involved in disease and cancer progression (Verma et al. 2018). In the active spliceosome, SNRNP25 heterodimerizes with other proteins in the complex, like SNRNP35 and programmed cell death 7 (PDCD7) (Zhao et al. 2025). According to the IMPC, homozygous knockout of *Pdcd7* results in an embryonic lethal phenotype before embryonic day 9.5 (Baldarelli et al. 2021). Although there are no reported mammalian knockouts, *Snrnp25*, like *Snrnp35* and *Pdcd7*, is expressed as early as the one-cell stage (Baldarelli et al. 2021), and knockdown of the gene in zebrafish, as well as knockdown of U12 splicing, leads to early embryonic death (König et al. 2007; Pei et al. 2018).

In conclusion, we have defined the boundaries that encompass the *Hba*^*b2(th)*^ deletion and confirmed that the coding sequence for *Hba-a2* is not deleted, thus demonstrating that the alpha-thalassemic phenotype observed in these mice is likely due to disruptions in regulatory sequences. Furthermore, we have identified *Snrnp25* as the likely gene essential for mammalian peri-implantation embryonic development within the *Hba* deletions.

## Electronic supplementary material

Below is the link to the electronic supplementary material.

Supplementary Material 1

## Data Availability

Data is provided within the manuscript or supplementary information files.
