# Supplementary material for: Snrnp25 is a candidate for the peri-implantation lethal phenotype of the Hba deletions
Source: Mamm Genome. 2025 May 21;36(3):727–34. doi: 10.1007/s00335-025-10133-z (PMC12408735; doi:10.1007/s00335-025-10133-z)
Supplement: Supplementary file 1 — Supplementary Material 1 [file 335_2025_10133_MOESM1_ESM.docx]

**Tables S1-4.** Amplicon alignment polymorphisms for each gene analyzed. Position based on amplicon sequence position (Data S1).

**S1.**

| ***Bod1 - Not Deleted*** | | | | |
| --- | --- | --- | --- | --- |
| **Position** | **B6** | **Cast** | **B6/Cast** | **Hba/Cast** |
| 52 | A | G | G | G |
| 54 | A | G | G | G |
| 274 | C | A | M | M |
| 344 | A | G | G | G |
| 397 | G | G | G | R |
| 421 | A | G | G | G |
| 445 | A | G | G | G |
| 453 | G | A | R | R |
| 523 | G | C | C | C |
| 542 | A | C | C | C |
| 622 | G | G | K | K |

**S2.**

| ***Cpeb4 - Deleted*** | | | | |
| --- | --- | --- | --- | --- |
| **Position** | **B6** | **Cast** | **B6/Cast** | **Hba/Cast** |
| 23 | A | G | G | G |
| 89 | T | T | C | T |
| 183 | C | T | Y | T |
| 294 | C | C | M | C |
| 385 | G | C | S | C |
| 518 | T | C | Y | C |
| 576 | T | A | W | A |
| 671 | G | A | A | A |
| 788 | T | C | C | C |

**S3.**

| ***Hba-a2 - Not Deleted*** | | | | |
| --- | --- | --- | --- | --- |
| **Position** | **B6** | **Cast** | **B6/Cast** | **Hba/Cast** |
| 140 | T | C | Y | Y |
| 197 | T | T | K | K |
| 321 | T | G | K | K |
| 329 | A | A | V | A |
| 364 | C | C | Y | Y |
| 411 | C | C | Y | Y |
| 503 | T | C | Y | Y |
| 605 | C | T | S | S |
| 638 | T | C | Y | C |

**S4.**

| ***Hbq1b - Deleted*** | | | |
| --- | --- | --- | --- |
| **Position** | **B6*** | **B6/Cast** | **Hba/Cast** |
| 111 | C | Y | T |
| 121 | C | M | A |
| 382 | T | W | A |
| 573 | A | R | G |
| *There is no CAST reference available for Hbq1b | | | |
